# The Quantitative Overhead Analysis for Effective Task Migration in Biosensor Networks

**DOI:** 10.1155/2013/965318

**Published:** 2013-09-26

**Authors:** Sung-Min Jung, Tae-Kyung Kim, Jung-Ho Eom, Tai-Myoung Chung

**Affiliations:** ^1^Department of Electrical and Computer Engineering, Sungkyunkwan University, 300 Cheoncheon-dong, Jangan-gu, Suwon-si, Gyeonggi-do 440-746, Republic of Korea; ^2^Department of Liberal Art, Seoul Theological University, Sosabon-dong, Sosa-gu, Bucheon-si, Gyeonggi-do 422-742, Republic of Korea; ^3^Department of Military Studies, Daejeon University, 62 Daehakro, Dong-Gu, Daejeon-si 300-716, Republic of Korea

## Abstract

We present a quantitative overhead analysis for effective task migration in biosensor networks. A biosensor network is the key technology which can automatically provide accurate and specific parameters of a human in real time. Biosensor nodes are typically very small devices, so the use of computing resources is restricted. Due to the limitation of nodes, the biosensor network is vulnerable to an external attack against a system for exhausting system availability. Since biosensor nodes generally deal with sensitive and privacy data, their malfunction can bring unexpected damage to system. Therefore, we have to use a task migration process to avoid the malfunction of particular biosensor nodes. Also, it is essential to accurately analyze overhead to apply a proper migration process. In this paper, we calculated task processing time of nodes to analyze system overhead and compared the task processing time applied to a migration process and a general method. We focused on a cluster ratio and different processing time between biosensor nodes in our simulation environment. The results of performance evaluation show that task execution time is greatly influenced by a cluster ratio and different processing time of biosensor nodes. In the results, the proposed algorithm reduces total task execution time in a migration process.

## 1. Introduction

**Figure 1:**
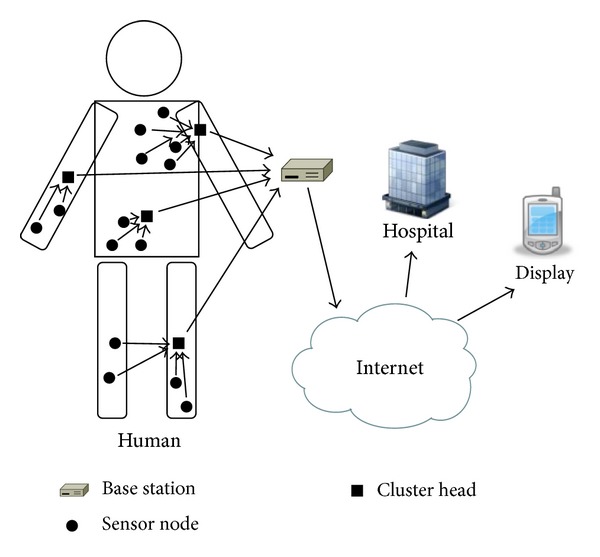
The concept of biosensor networks.

A biosensor network is generally composed of many biosensor nodes and one base station. Biosensor nodes are distributed on a human body or wearable devices, and the base station is located at outside of biosensor networks. Biosensor nodes monitor various biological parameters such as body temperature, blood pressure, and blood glucose level. They transmit these gathered data to the base station, and the base station derives meaningful results from the processed data. Finally, the base station sends these results to user's device or to a hospital through the internet as shown in Figure 1 [1].

In general, main components of a biosensor node are a sensing unit, a processing unit, a transceiver, and a power unit [2, 3]. Biosensor nodes can monitor specific parameters by using sensing units, and gathered information is delivered to the processing unit. The processing unit is composed of a processor, storage, and memory. These subunits manage a procedure to analyze collected information and relay it to other biosensor nodes. The collected information by biosensor nodes is typically medical data, so it is sensitive and privacy information. This information should not be exposed to a malicious user, and biosensor nodes should process it in real time [4, 5]. Because of their small size, biosensor nodes have little computational power, limited capacity of memory, and restricted battery. Thus, an attack can easily decrease availability of biosensor nodes, so it makes it impossible that they relay the important information to user in real time [6, 7]. In this case, a suitable migration process has to be used to solve that problem. The migration means the process of transferring tasks from nodes with heavy overhead to other nodes with enough capabilities.

In this paper, we propose a useful algorithm to quantitatively analyze system overhead. Simulation results show that network performance is greatly influenced by a cluster ratio and different performance of biosensor nodes. Also, using the proposed algorithms, the total task execution time is reduced compared with a general process. The remainder of this paper is organized as follows. In Section 2, we discuss restricted resources of biosensor nodes and the reason to use a clustering scheme in our system model. In Section 3, we present mathematical analysis to calculate total task execution time and system overhead. In Section 4, we evaluate the proposed algorithm with several parameters. Finally, Section 5 concludes this paper.

## 2. The Clustering Scheme in System Model

**Figure 2:**
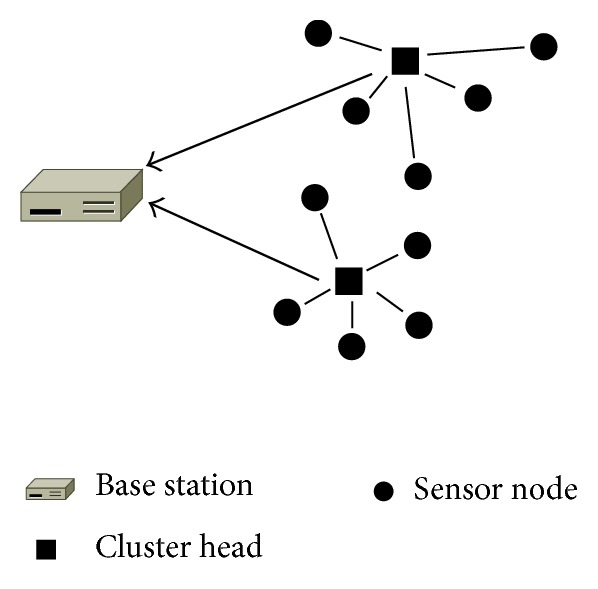
The clustering scheme.

Biosensor nodes are generally very small, so the use of computing resources is limited. In particular, it is impossible to replace or recharge the power unit, so it is important to reduce energy consumption in the biosensor network. Since it is necessary to make uniform energy consumption to all biosensor nodes, we need to use a hierarchical routing protocol [8, 9]. This protocol uses a cluster which indicates a logical group of biosensor nodes, and the cluster is managed by the leader node called a cluster head. Before biosensor nodes gather data, cluster heads are selected, and clusters are formed around these cluster heads in the hierarchical routing protocol. The cluster heads are responsible for gathering information from all biosensor nodes in their cluster. After gathering information, cluster heads perform data aggregation to reduce data size and transmit results to the base station. The role of the cluster head is periodically rotated to prevent energy depletion of particular biosensor nodes. Therefore, our system model uses the hierarchical routing protocol to reduce energy consumption.  Figure 2 shows the clustering scheme.

Biosensor nodes are generally distributed in wearable equipment or on a human body. It is assumed that the base station knows the network topology. Also, the base station has sufficient battery and processing capability. There are two kinds of sensors such as cluster heads and normal biosensor nodes. Biosensor nodes have formed the cluster by using the cluster head selection algorithm [10, 11]. When overhead is occurs in some nodes, a suitable migration process could be used to reduce overhead. In other words, tasks are moved from biosensor nodes with large overhead to the other nodes with sufficient resources. The task execution time can represent system overhead, so we calculate and compare it to analyze system overhead in our system model.

First, we set that task execution time is the sum of processing time and communication time as shown in (1). Processing time indicates the time required to process the tasks in each biosensor node. Thus, total processing time of all tasks depends on the number of active biosensor nodes. Communication time means the time required to transmit from each biosensor nodes to the base station. There are two types of communication time in the hierarchical routing protocol. One is the transmission time from biosensor nodes to cluster heads, and the other is transmission time from cluster heads to the base station:

(1) Task  execution  time=Processing  Time   +Communication  Time.

**Figure 3:**
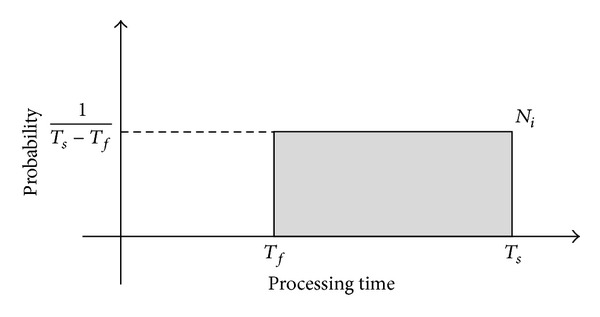
The probability density function.

The biosensor nodes check processing time in regular period and record the fastest and slowest value. Let *T*_*f*_ denote the fastest processing time and let *T*_*s*_ denote the slowest processing time which is required to process a unit task. It is assumed that the processing time follows uniform distribution from *T*_*f*_ to *T*_*s*_. Figure 3 shows its probability density function. Let *N*_*i*_ denote the initial number of biosensor nodes to process all the tasks in this function.

In this function, the expected value is as shown in (2) by a uniform distribution rule. If there is no consideration of a migration process to calculate the task execution time, then (2) is used to calculate the task execution time of biosensor nodes:

(2) $$\begin{array}{c} E\left[X\right]=\frac{T_{f}+T_{s}}{2}. \end{array}$$

## 3. The Proposed Algorithm to Analyze System Overhead

We focus on the number of biosensor nodes and assume that the system performance is linearly improved by the number of nodes [12]. Thus, the number of active biosensor nodes is different by system overhead. When some biosensor nodes have heavy overhead, we can solve this problem to use a migration process. Let *N*_*i*_ denote the initial number of biosensor nodes. After we move tasks from *N*_*w*_/*N*_*i*_ of nodes to the other nodes, the number of active biosensor nodes becomes *N*_*w*_.

**Figure 4:**
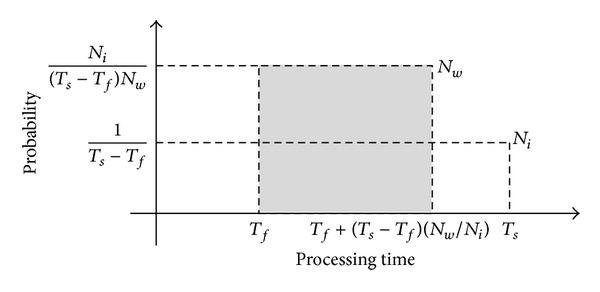
The new probability density function.

As a result, the processing time becomes a new uniform distribution as shown in Figure 4. It is distributed from *T*_*f*_ to *T*_*f*_ + (*T*_*s*_ − *T*_*f*_)(*N*_*w*_/*N*_*i*_). Equation (3) shows the expected value of its probability density function:

(3) $$\begin{array}{c} E\left[X\right]=T_{f}+\frac{\left(T_{s}-T_{f}\right)}{2N_{i}}\cdot N_{w}. \end{array}$$

We should consider the number of tasks to calculate system overhead. Let *N*_*e*_ denote the number of tasks. Since the number of active biosensor node is *N*_*w*_, each biosensor node has to process *N*_*e*_/*N*_*w*_ tasks. Equation (4) indicates the expected value of processing time in each biosensor node:

(4) $$\begin{array}{c} T_{\text{process}}=\frac{N_{e}}{N_{w}}\left(T_{f}+\frac{\left(T_{s}-T_{f}\right)}{2N_{i}}\cdot N_{w}\right). \end{array}$$

As mentioned in the previous section, our system model uses a clustering scheme, and the number of biosensor nodes is similar in each cluster. Let *R*_*c*_ denote cluster ratio; then, the number of clusters will be *N*_*w*_ × *R*_*c*_, and the number of biosensor nodes in one cluster will be 1/*R*_*c*_.

**Figure 5:**
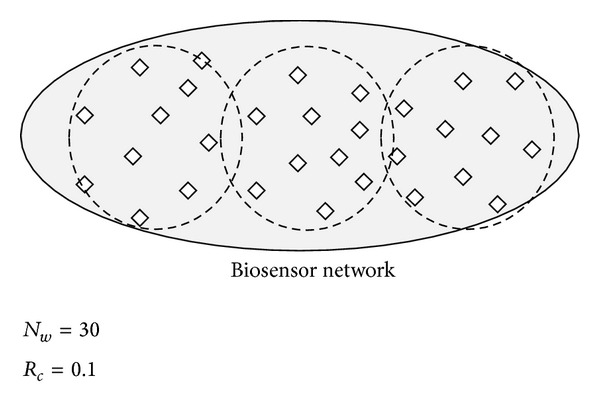
The number of cluster by a cluster ratio.

For example, the number of biosensor nodes is 30, and the cluster ratio is 0.1; then, the number of clusters is 3 (30 × 0.1), and the number of biosensor nodes is 10 (1/0.1) in each cluster as shown in Figure 5.

**Figure 6:**
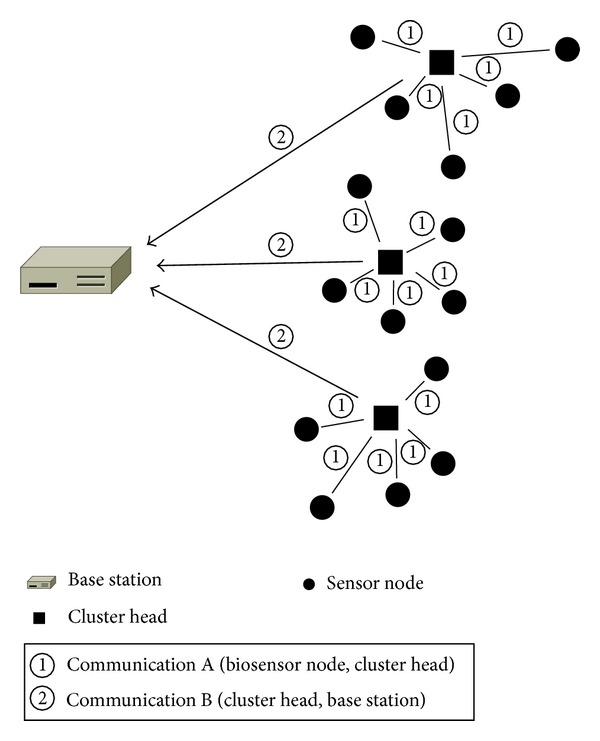
The two types of communication.

We calculate the communication time in the biosensor network. Let *T*_*t*_ denote transmission time of unit packet. There are two types of communication as shown in Figure 6. First, the communication time from a biosensor node to a cluster head in each cluster is represented as the product of the data transmission time and the number of biosensor nodes in each cluster. Biosensor nodes sequentially transmit results according to the order, and the communication time from biosensor nodes to a cluster head can be presented as *T*_*t*_ × (1/*R*_*c*_). Second, the communication time from a cluster head to the base station is expressed as the product of data transmission time and the number of cluster heads. Because cluster heads also sequentially send results to the base station, communication time in second case is expressed as *T*_*t*_ × *N*_*w*_ × *R*_*c*_. Equation (5) indicates the total communication time:

(5) $$\begin{array}{c} T_{\text{communication}}=T_{t}\cdot \frac{1}{R_{c}}+T_{t}\cdot N_{w}\cdot R_{c}. \end{array}$$

Finally, the sum of (4) and (5) represented the time required to process all tasks and transmit to the base station when the number of active biosensor nodes is changed from *N*_*i*_ to *N*_*w*_:

(6) $$\begin{array}{c} T_{\text{all}}=\frac{N_{e}}{N_{w}}\left(T_{f}+\frac{\left(T_{s}-T_{f}\right)}{2N_{i}}\cdot N_{w}\right)+T_{t}\cdot \frac{1}{R_{c}}+T_{t}\cdot N_{w}\cdot R_{c}. \end{array}$$

## 4. Performance Evaluation

We evaluate the total task execution time by (6) in a biosensor network. We use the parameter values listed in Table 1 for our analysis of task execution time. We set the number of tasks (*N*_*e*_) to 100 and the initial number of biosensor nodes (*N*_*i*_) to 30. The number of active biosensor nodes (*N*_*w*_) is from 1 to 30. The fastest processing time of a biosensor node (*T*_*f*_) is 0.001 seconds. We set the unit message length to 128 byte and transmission speed to 250 kbps. Therefore, it takes about 0.0041 seconds to process the unit message length so we set the transmission time (*T*_*t*_) to 0.0041 seconds. Since the communication range of a biosensor network is very small and there is very little impact on the performance by distance between biosensor nodes, the distance is ignored in our performance evaluation.

Based on these simulation parameters, we evaluate total task execution time according to change of a cluster ratio and the slowest processing time of a biosensor node in a migration process.

**Figure 7:**
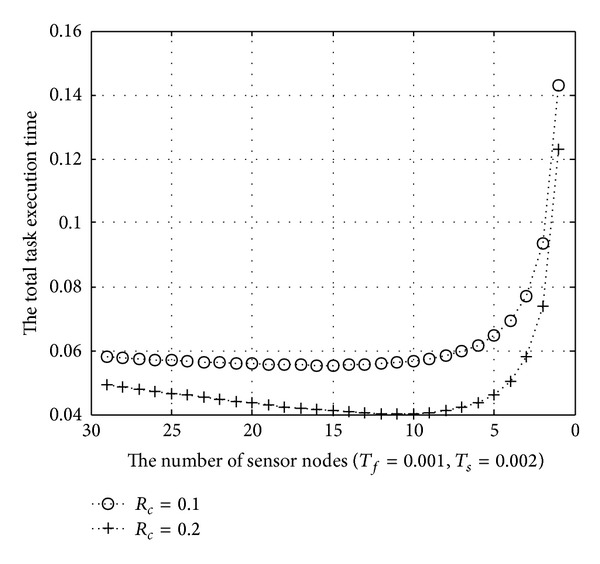
The total task execution time (*T*_*s*_ = 0.002).

**Figure 8:**
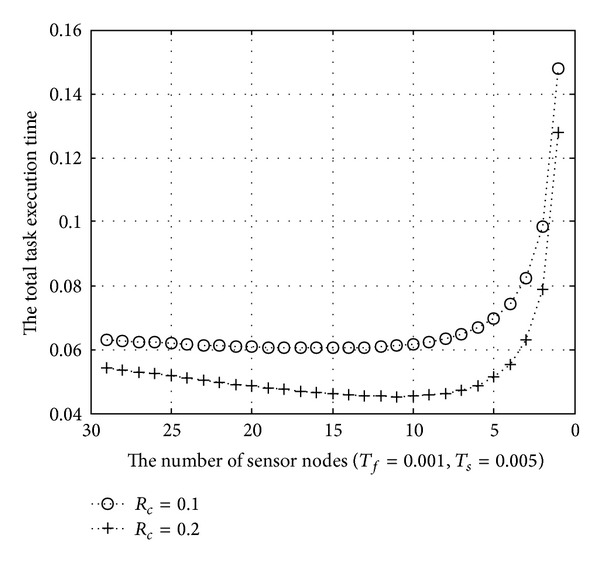
The total task execution time (*T*_*s*_ = 0.005).

In Figures 7 and 8, we set *T*_*s*_ to 0.002 and 0.005 seconds, respectively. Also, we calculate task execution time according to the change of a cluster ratio (*R*_*c*_). *R*_*c*_ is 0.1 and 0.2 in each figure.  Figure 7 shows the result between the total task execution time and *N*_*w*_. In this evaluation, *T*_*f*_ is set as 0.001, and *T*_*s*_ is set as 0.002 seconds. At first, the total task execution time decreases as the number of active biosensor nodes decreases. However, the task execution time increases after a certain number of biosensor nodes. It is 15 in case *R*_*c*_ is 0.1 and 10 in case *R*_*c*_ is 0.2 in Figure 7.

Figure 8 shows the result when *T*_*f*_ is 0.001 and *T*_*s*_ is 0.005. We can recognize that total task execution time is influenced by *T*_*s*_ as compared with the result of Figure 7. Overall, the task execution time also increases as the different processing time increases. After the number of biosensor nodes becomes about 30% of the initial number of them, the task execution time increases rapidly. Thus, we can know that system overhead is tolerable until this point. As the cluster ratio increases, the change of the total task execution time decreases. As shown in the graph in Figures 7 and 8, total task execution time is affected by a cluster ratio and difference of processing time among biosensor nodes. Thus, we have to control these parameters to manage biosensor networks efficiently.

When biosensor nodes have heavy overhead, we can solve this problem by moving tasks from these nodes to other nodes with enough resources. The number of active biosensor nodes is changed, and it is needed to accurately calculate overhead in biosensor network. Also, we evaluate the total task execution time by (6) as the change of the slowest processing time of biosensor nodes.

**Figure 9:**
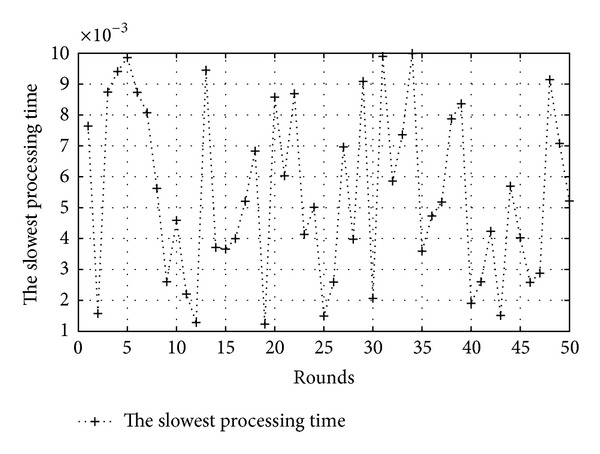
The slowest processing time (*T*_*f*_ = 0.001).

Figure 9 shows the change of the slowest processing time in our simulation. There are different values from 0.001 to 0.010 seconds. We set *T*_*f*_ to 0.001 seconds. At each round we compared the task execution time applied to migration scheme and the task execution time applied to general method.

**Figure 10:**
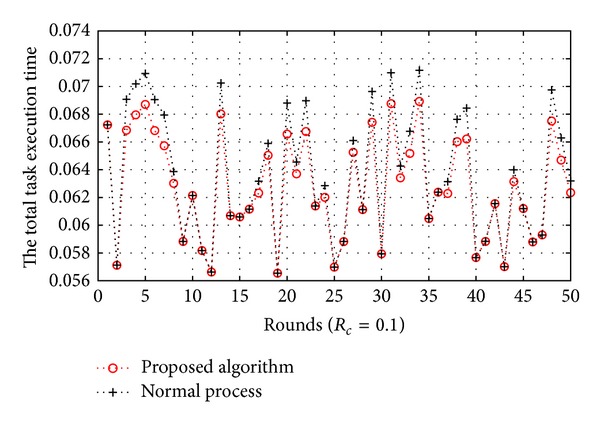
The total task execution time (*R*_*c*_ = 0.1).

Figure 10 shows that the total execution time as *T*_*s*_ is changed when *R*_*c*_ is 0.1. The number of active nodes is 30. If *T*_*s*_ is greater than or equal to nine times of *T*_*f*_, then tasks in 30% of all biosensor nodes move to the other biosensor nodes in our proposed algorithm. In the same way, *T*_*s*_ is greater than or equal to seven times and five times of *T*_*f*_, and we move the tasks in 20% and 10% of all biosensor nodes, respectively. In Figure 10, the sum of execution time applied to normal process at each round is 3.1795 seconds, and the sum of execution time applied to a migration process is 3.1372 seconds. We can reduce the total task execution time by using our proposed algorithm, and the system performance has been improved by 1.35% in this case.

**Figure 11:**
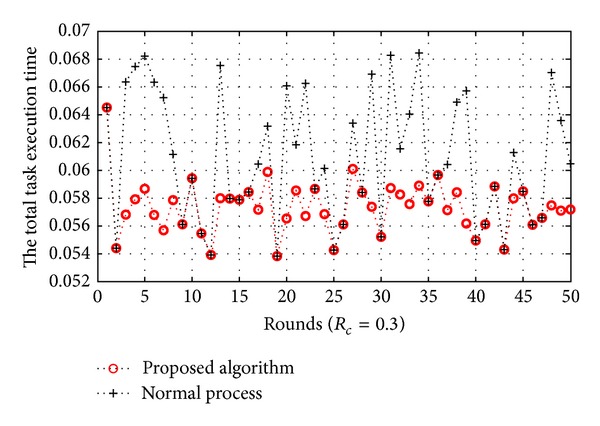
The total task execution time (*R*_*c*_ = 0.3).

Figure 11 shows the result between the total execution time and *T*_*s*_ at each round when *R*_*c*_ is 0.3. If we do not consider a migration process, the total execution time is 3.0440 seconds. Conversely, if we use our proposed algorithm, the total execution time is 2.8677 seconds. Also, we can decrease the total execution time, and the system performance has been improved by 6.15% in this case.

When some biosensor nodes have large overhead, proper migration process is needed to manage the biosensor network efficiently. We suggest the algorithm to quantitatively analyze the total task execution time for effective task migration. The proposed algorithm is useful to apply a proper migration process, and the simulation result shows that it efficiently reduces the total task execution time.

## 5. Conclusion

A biosensor network is composed of many biosensor nodes with sensing, computation, and wireless communication capabilities to collect biological parameters of a human body. Biosensor nodes collect these parameters and relay them to other biosensor nodes or to the base station. Biosensor nodes have restricted resources due to their small size. Thus, the biosensor network is vulnerable to an external attack. When the malicious user attacks the system, some nodes have heavy overhead and the overall system performance will be degraded. We can solve this problem to apply a proper migration process.

In this paper, we propose the quantitative solution to figure out task execution time. Also, we compare the total task execution time applied to a migration process and a general method. The results of performance evaluation show that total execution time is affected by a cluster ratio and processing time between biosensor nodes. Therefore, it is needed to manage a cluster ratio and difference of processing time against an attack. Our proposed algorithm reduces the total task execution time by using a proper migration process. In this scheme, the method to calculate the processing time of biosensor nodes is not considered. Therefore, we are going to research to accurately calculate the processing time for more accurate simulation.

## Figures and Tables

**Table 1:** Parameters for analysis.

Parameters	Values	Descriptions
*N*_*e*_	100	The number of tasks
*N*_*w*_	1 ~ 30	The number of active biosensor nodes
*R*_*c*_	0.1, 0.2, 0.3	The cluster ratio
*T*_*f*_	0.001	The fastest processing time of a biosensor node
*T*_*s*_	Variable	The slowest processing time of a biosensor node
*N*_*i*_	30	The initial number of biosensor nodes
*T*_*t*_	0.0041 sec	The transmission time
